# Correction: Felix, L.; Ziemens, D.; Seifert, G.; Rose, C.R. Spontaneous Ultraslow Na^+^ Fluctuations in the Neonatal Mouse Brain. *Cells* 2020, *9*, 102

**DOI:** 10.3390/cells9112380

**Published:** 2020-10-30

**Authors:** Lisa Felix, Daniel Ziemens, Gerald Seifert, Christine R. Rose

**Affiliations:** 1 Institute of Neurobiology, Faculty of Mathematics and Natural Sciences, Heinrich Heine University, 40225 Duesseldorf, Germany; Lisa.Felix@hhu.de (L.F.); Daniel.Ziemens@hhu.de (D.Z.); 2 Institute of Cellular Neurosciences, Medical Faculty, University of Bonn, D-53105 Bonn, Germany; Gerald.Seifert@ukbonn.de

The authors wish to make the following changes to their paper [1].

We regret to have to inform the reader about a series of errors which relate to the control data set, which describes the basic observation of spontaneous ultraslow Na^+^ fluctuations in the hippocampus of neonates. This error arose because the authors originally split this data set of controls, but later on decided that the full set of data would be put into the manuscript, thereby increasing the number of control observations. Unfortunately, in the entire text as well as in Table 2 and Figure 1C, Figure 3B, and Figure 4A,B, the authors mistakenly reported the control values for one of the split data sets.

In the Abstract, “we found that 20% of pyramidal neurons and 44% of astrocytes” should be replaced with “…we found that 22% of pyramidal neurons and 43% of astrocytes…”.

In Section 3.1, in the first paragraph, “…were present in 44% of recorded astrocytes (*n* = 51/116 cells; Table 2) and in 20% of recorded neurons (*n* = 49/234 cells; Table 2, Figure 1C). The frequency of these fluctuations was very low, with the active astrocytes averaging at 1.8 ± 1.1 fluctuations/hour and the active neurons exhibiting 1.5 ± 0.9 fluctuations/hour.” should be replaced with “…were present in 43% of recorded astrocytes (*n* = 92/213 cells; Table 2) and in 22% of recorded neurons (*n* = 89/412 cells; Table 2, Figure 1C). The frequency of these fluctuations was very low, with the active astrocytes averaging at 1.8 ± 1.2 fluctuations/hour and the active neurons exhibiting 1.9 ± 1.3 fluctuations/hour”.

In the third paragraph, “…average duration of 8.7 ± 3.4 min in astrocytes (*n* = 91 fluctuations; Table 2) and 9.2 ± 4.2 min in neurons (*n* = 116 fluctuations; Table 2). The mean amplitude for fluctuations was 2.1 ± 1.3 mM and 2.2 ± 1.2 mM for astrocytes…” should be replaced with “…average duration of 8.6 ± 3.2 min in astrocytes (*n* = 166 fluctuations; Table 2) and 9.2 ± 4.1 min in neurons (*n* = 166 fluctuations; Table 2). The mean amplitude for fluctuations was 2.1 ± 1.4 mM and 2.2 ± 1.2 mM for astrocytes…”.

In Section 3.2, in the second paragraph, “, dropping in astrocytes from 44% (*n* = 116) in neonatal animals…” should be replaced with “…dropping in astrocytes from 43% (*n* = 213) in neonatal animals…”; “, starting at 20.1% in neonates (*n* = 234) and staying at” should be replaced with “, starting at 21.6% in neonates (*n* = 412) and staying at…”. In the third paragraph, “…mean frequency (signals/hour) of 1.8 ± 1.1, 1.7 ± 0.8, 1.4 ± 0.7, and 1.1 ± 0.3 for astrocytes and 1.5 ± 0.9, 2.6 ± 1.6…” should be replaced with “a mean frequency (signals/hour) of 1.8 ± 1.2, 1.7 ± 0.8, 1.4 ± 0.7, and 1.1 ± 0.3 for astrocytes and 1.9 ± 1.3, 2.6 ± 1.6”.

In Section 3.5, in the first paragraph, “…we observed an increase in astrocyte amplitudes (2.9 ± 1.8 mM; *p* < 0.001).” should be replaced with “we observed an increase in astrocyte amplitudes (2.9 ± 1.8 mM; *p* < 0.002)”.

Correspondingly, Figure 1 should be replaced with the following:

**Figure 1:**
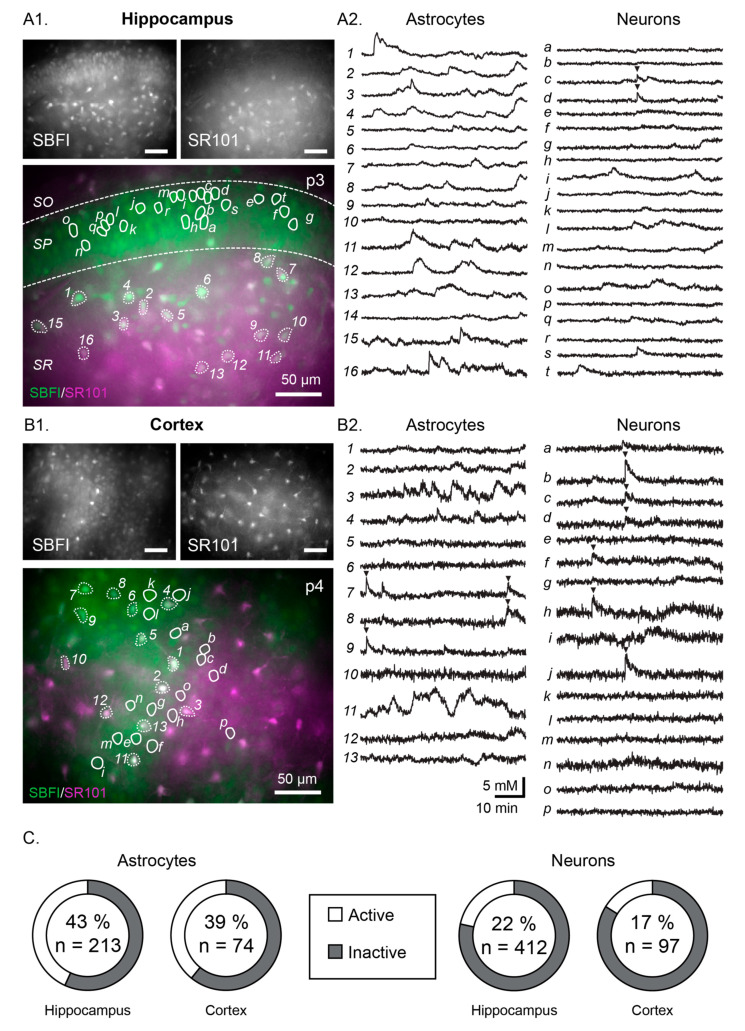
Example measurements showing spontaneous Na^+^ fluctuations in the neonate hippocampus (**A1**,**A2**) and neocortex (**B1**,**B2**). (**A1**,**B1**) show the SBFI (top left), SR101 (top right) and merge images (bottom) with all scale bars indicating 50 µm. Circled areas correspond to regions of interest (ROIs), the individual fluorescent measurement traces of which are illustrated in (**A2**,**B2**) (astrocytes on the left and numbered, neurons on the right and labelled with letters). Arrows indicate instances when the cells appear to be synchronized. (**C**) Pie charts indicating the percentage of active astrocytes (left) and neurons (right) within each area (*n* = total number of cells measured). SBFI: sodium-binding benzofuran isophthalate-acetoxymethyl ester.

Table 2 should be replaced with the following:

**Table 2:** Number of neurons (left) and astrocytes (right) measured, the % of these showing activity, the total number of fluctuations analyzed under each condition. and the mean and standard deviation for the amplitude (mM) and duration (minutes) for the analyzed fluctuations.

	Neurons								Astrocytes							
	Cells (n)	% Cells (n) Active	Fluctuation (n)	mM Mean	mM SD	*p* Values	Min Mean	Min SD	Cells (n)	% Active	Fluctuation (n)	mM Mean	mM SD	*p* Values	Min Mean	Min SD
**Control**	**412**	**21.6**	**166**	**2.2**	**1.2**		**9.2**	**4.1**	**213**	**43**	**166**	**2.1**	**1.4**		**8.6**	**3.2**
**TTX**	**94**	7.4	7	**0.7**	**0.3**	**	8.2	3.7	**39**	35.9	23	**1**	**0.3**	***	8.7	4.1
**BIC**	**76**	19.7	32	**0.8**	**1**	***	6.6	2.3	**22**	59.1	20	2.7	1.9	0.06	8.1	2.3
**CGP**	**101**	12.9	20	2	0.6	0.48	10.9	3.5	**49**	44.9	42	2.1	1.2	0.78	9.6	4.6
**SNAP/NNC**	**53**	22.6	17	**1.1**	**0.2**	***	10.2	3.1	**86**	34.9	33	**3.2**	**2.0**	**	10.8	6.2
**NPA**	**70**	15.7	20	**1**	**0.3**	***	10.7	3.8	**43**	32.6	31	**1.3**	**0.6**	**	9.4	3.6
**Sarcosine**	**74**	20.3	20	1.5	0.5	0.02	11.3	3.7	**62**	21	25	2.6	1.7	0.06	8.5	3.5
**TFB-TBOA**									**36**	27.8	10	1.9	1.7	0.69	8.5	3.7
**CNQX**	**130**	6.2	9	2.6	1.3	0.29	9.6	4.1	**45**	37.8	25	2.1	1.1	0.86	8.9	3.1
**APV**	**156**	10.3	19	2.5	0.9	0.22	10.9	5.2	**52**	28.8	19	2.7	2.5	0.34	13.1	4.9
**MPEP**	**117**	16.2	30	1.9	0.7	0.28	9.5	3.8	**26**	46.2	53	3.9	2.2	0.36	9.1	6.2
**α-BT**	**118**	11.9	29	1.6	0.6	0.02	10.1	3.7	**43**	67.4	89	2	1.5	0.93	7.3	3.7
**Atropine**	**124**	16.1	28	2.6	1.3	0.14	8.5	2.9	**73**	49.3	74	2.5	1.2	0.02	7.7	2.9
**PPADs**	**70**	11.4	19	1.4	0.9	0.01	10.7	4.9	**57**	24.6	24	1.6	0.6	0.1	8	2.4
**Caffeine**	**142**	22.5	75	1.9	0.7	0.05	9.4	4.8	**32**	62.5	33	2	0.9	0.84	8.8	3.2
**Prazosin**	**111**	13.5	21	**1.3**	**0.4**	**	9.7	3.7	**81**	40.7	50	1.7	0.8	0.18	10.4	4.6
**Propranolol**	**123**	7.3	11	1.9	0.5	0.55	11.4	4.3	**59**	38.9	54	2.7	1.2	0.01	8.7	4.1
**SEA0400**	**85**	11.7	12	2	1.3	0.65	8.8	3.1	**18**	44.4	9	2.6	1	0.21	8	1.4
**Bumet**	**132**	6.1	8	1.8	0.5	0.44	11	3.1	**29**	37.9	15	1.6	1.3	0.28	8.5	3.5
**Amiloride**	**162**	11.1	31	1.8	0.6	0.11	8.7	2.7	**50**	28	20	1.7	0.5	0.34	8.8	3.5
**La^3+^**	**144**	5.2	8	1.8	0.6	0.43	11.9	5.3	**84**	40.5	74	2.0	1.2	0.71	8.4	3.4
**C57Bl/6 WT**	**460**	11.1	66	1.5	0.8		10.8	4.9	**548**	51.1	620	2.1	1.5		8.7	4.0
**Cx -/-**	**77**	18.2	30	1.6	0.6	0.65	11.4	5.6	**46**	60.1	58	2.2	1.5	0.77	8.7	4.3

Abbreviations, see Table 1. Bumet: bumetanide; a-BT: a-bungarotoxin. Red color indicates statistically significant difference as compared to the control condition, *p*-values indicated apply to the amplitudes; ** 0.001 ≤ *p* < 0.01, *** *p* < 0.001.

Figure 3 should be replaced with the following:

**Figure 3:**
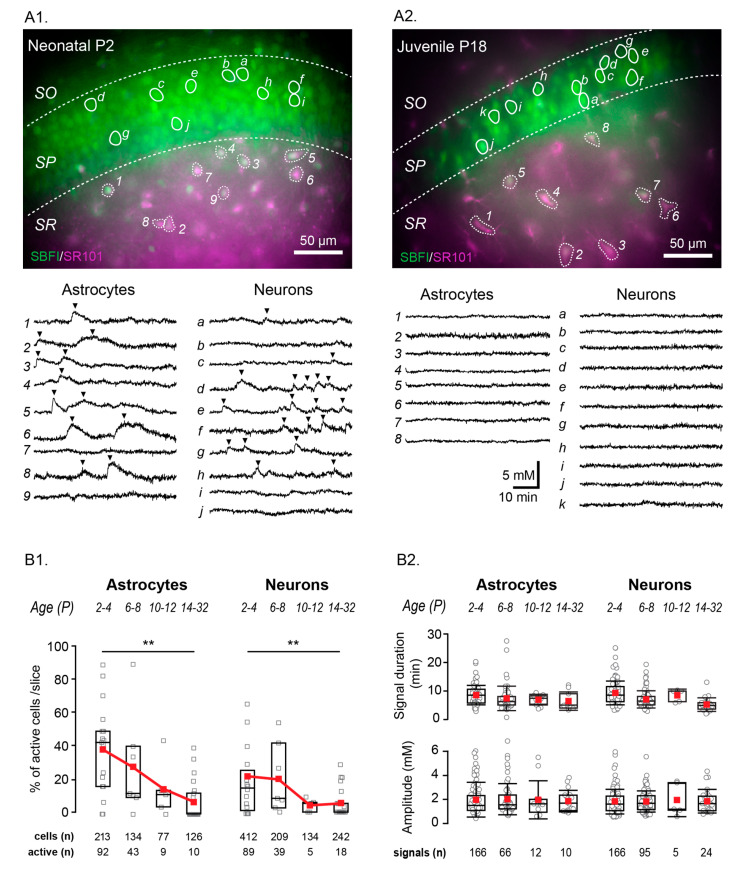
Age dependence of spontaneous Na^+^ fluctuations. Example merged staining of a P2 (**A1**) and P18 (**A2**) hippocampal slice (SBFI in green, SR101 in magenta, overlapping regions in white) with example cells encircled and their individual fluorescent measurement traces shown below. Analyzed fluctuations are indicated by black arrows. Scale bars show 50 µm. SO, SP, SR: stratum oriens, pyramidale, and radiatum, respectively. (**B1**) Box plots illustrating the decline in the percentage of cells showing activity per slice with increasing mouse age. (**B2**) Properties from fluctuations in different age groups, as written above. Box-and-whisker plots indicate the median (line), mean (red square), interquartile range (box), and standard deviation (whiskers). ** 0.001 ≤ *p* < 0.01.

Figure 4 should be replaced with the following:

**Figure 4:**
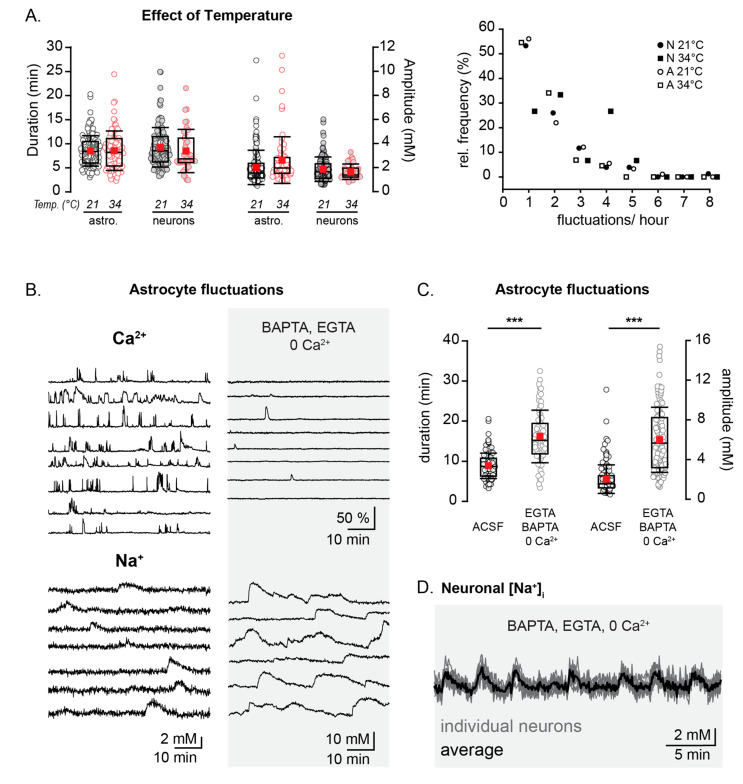
Temperature dependence and interrelation with spontaneous Ca^2+^-signaling. (**A**) Left: the duration and amplitude of neonatal astrocytic and neuronal Na^+^ fluctuations at 21 °C (black) and 34 °C (red). Right: the relative frequency distribution plot of neonatal Na^+^ fluctuations at 21 °C (neurons- black circles, astrocytes- white circles) and 34 °C (neurons- black squares, astrocytes- white squares). (**B**) Example traces from individual astrocytes in Ca^2+^ imaging experiments (top) and during Na^+^ imaging (bottom), both in ACSF (left) and in the presence of a Ca^2+^-chelated solution (0 Ca^2+^, ACSF containing 500 µM of BAPTA-AM and 1 mM of EGTA) (right). (**C**) Box plots illustrating the increase in the astrocytic Na^+^ fluctuation amplitudes after the chelation of Ca^2+^. (**D**) Traces showing the Na^+^ fluctuations in several individual neurons (grey) and an averaged trace (black) in a Ca^2+^-chelated solution. *** *p* < 0.001.

Figure 5 should be replaced with the following:

**Figure 5:**
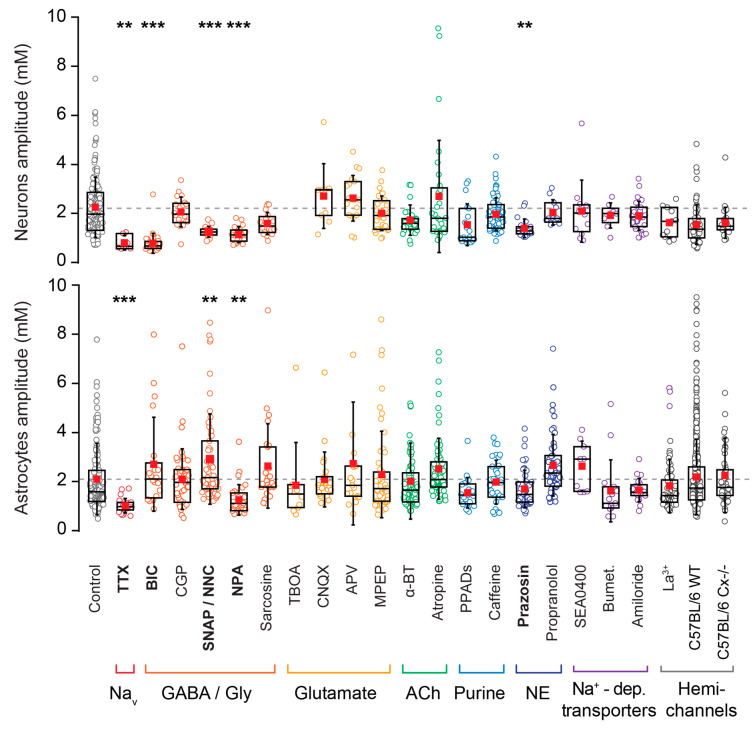
Pharmacological investigation. Comparison of the fluctuation amplitudes in neurons and astrocytes in the presence of various pharmacological conditions, as detailed in Table 1 and Table 2. Blockers are arranged into color groups according to their target pathway, as indicated below the plots. Abbreviations, see Table 1. Bumet: bumetanide; a-BT: a-bungarotoxin. One data point (10.95 mM) from the astrocytic control group is not shown here. ** 0.001 ≤ *p* < 0.01, *** *p* < 0.001.

The authors would like to apologize for any inconvenience caused to the readers by these changes. The changes do not affect the scientific results. The manuscript will be updated and the original will remain online on the article webpage, with a reference to this correction.
